# Biocontrol potential of lipopeptides produced by *Paenibacillus polymyxa* AF01 against *Neoscytalidium dimidiatum* in pitaya

**DOI:** 10.3389/fmicb.2023.1188722

**Published:** 2023-05-17

**Authors:** Shanyu Lin, Xiaohang Chen, Ling Xie, Yan Zhang, Fenghua Zeng, Yanyan Long, Liyun Ren, Xiuling Qi, Jiguang Wei

**Affiliations:** ^1^College of Agriculture, Guangxi University, Nanning, China; ^2^Key Laboratory of Green Prevention and Control on Fruits and Vegetables in South China Ministry of Agriculture and Rural Affairs, Guangxi Key Laboratory of Biology for Crop Diseases and Insect Pests, Plant Protection Research Institute, Guangxi Academy of Agricultural Science, Nanning, Guangxi, China; ^3^Baise Agricultural Scientific Research Institute, Baise, China; ^4^College of Agricultural Engineering, Guangxi Vocational University of Agriculture, Nanning, China

**Keywords:** *Paenibacillus polymyxa*, *Neoscytalidium dimidiatum*, pitaya canker, fusaricidins, RNA-Seq

## Abstract

Pitaya canker, caused by *Neoscytalidium dimidiatum*, is one of the most important fungal diseases that cause significant losses in production. To replace chemical pesticides, the use of biocontrol strains to manage plant diseases has been the focus of research. In this study, the bacterial strain AF01, identified as *Paenibacillus polymyxa*, exhibited significant antifungal effects against *N. dimidiatum* and four other pitaya fungal pathogens. The strain *P. polymyxa* AF01 produces 13 fusaricidins, which directly inhibit mycelial growth, spore germination and germ tube elongation by causing the membrane integrity and cell ultrastructure to incur irreversible damage. Pot experiment and yield test confirmed that AF01 provided preservative effects by reducing the disease index. In comparison to the untreated control groups, RNA-seq data showed that *P. polymyxa* AF01 selectively blocked some transcription and translation processes and inhibited RNA and DNA structural dynamics, energy production and conversion, and signal transduction, particularly cell wall biosynthesis, changes in membrane permeability, and impairment of protein biosynthesis. Thus, *P. polymyxa* AF01 could be potentially useful as a suitable biocontrol agent for pitaya canker.

## 1. Introduction

Pitaya (*Hylocereus* spp.), also known as dragon fruit, is a tropical and subtropical fruit native to Latin America, including Mexico, Central America, and tropical South America. Currently, pitaya cultivation is concentrated in Australia, China, Southeast Asia, Malaysia, Vietnam, Thailand, and the Philippines (Adnan et al., 2011). Pitaya contains numerous nutrients such as vitamins, minerals, fiber, protein, flavonoids, betanin, and polyphenols. Additionally, the beneficial effects of red pitaya on several human diseases have been reported (Ramli et al., 2016). Recently, canker caused by *Neoscytalidium dimidiatum* has become one of the most serious diseases affecting pitaya (Hong et al., 2020). The disease progression in pitaya plants begins with tiny dimples on the infected cladode; then, chlorotic spots develop and gradually turn yellow, even brown, and form flat, raised, hard brown scabs. As the disease progresses, the lesions develop a typical light-gray coloration with numerous dark spots. Under high-humidity conditions, lesions can cause softening and dissolution of tissues and structures, leading to the formation of cavities and collapse (Chuang et al., 2012). The *N. dimidiatum* fungus produces two types of conidia: one occurring in arthric chains in aerial mycelium or in ostiolate pycnidia embedded on the surface of mature lesions, and the other type is ellipsoid to nearly fusiform, hyaline even dark brown (Crous et al., 2006; Phillips et al., 2013). Conidia are difficult to eradicate and remain viable for several years. When diseased cladodes exist in orchard soil or drains, the lesions can continue to produce spores and infect young growth (Fullerton et al., 2018). If not controlled effectively, pitaya cankers can destroy the canopy, disfigure the fruit, and render orchards uneconomical. Current management strategies for pitaya canker, include the use of disease-resistant cultivars and chemical fungicides. However, there are several limitations to developing new disease-resistant cultivars to control pitaya canker, such as variations in cultivar resistance and differences in isolate virulence (Harsonowati et al., 2020). Therefore, farmers were forced to apply excessive doses and costly fungicide treatments throughout the infection season as susceptible cultivars grew. However, the frequent use of chemical pesticides results in high levels of pathogen resistance, pesticide residues, and environmental pollution, thereby becoming a human health concern (De Miccolis Angelini et al., 2014).

Considering these problems, new measures, such as the use of microbial fungicides, are desirable to control the disease and reduce the adverse effects of chemical fungicides. Biological control is one of the most promising and safe measures to suppress diseases in plants (Choudhary and Johri, 2009; Wu et al., 2022). For example, the adaptable metabolism, rapid growth, and high mobility of fluorescent pseudomonads make them valuable in plant disease control (Bakker et al., 2007; Du et al., 2017). Similarly, metabolites from *Bacillales* species activate plant immune regulators (Ryu et al., 2004). In general, antifungal substances produced by antagonistic bacteria have been reported to be the most important mechanism of inhibition of pathogen growth as it affects the lipid metabolism, amino acid metabolism, carbohydrate metabolism, membrane transport, and energy metabolism (Zuo et al., 2017). These antifungal substances had been identified as antagonistic proteins, enzymes and bacteriocins synthesized by the ribosomal pathway. Other antifungal substances include lipopeptides, polyketides, peptides, and volatile antifungal substances. With a broad range of antibiotic activity and low toxicity, cyclic lipopeptides (CLPs) synthesized by *Bacillus* spp. (iturins, surfactin, and fengycins, etc.), *Pseudomonas* spp. (massetolide A and putisolvin, etc.) and *Paenibacillus* spp. (fusaricidins, polymyxins) have been the focus of research in the control of plant diseases in recent years (Mülner et al., 2020; Soni et al., 2021). In nature products discovery, matrix-assisted laser desorption/ionization time-of-flight mass spectrometry (MALDI-TOF MS), have become in crucial for the identification of microorganisms and chemotyping of secondary metabolites (Mülner et al., 2021). Compounds were detected from culture supernatants or surface extracts of colonies without the need for further purification prior to mass spectrometric analysis. This technique affords simple preparation and efficient investigation of complex families of natural compounds, such as the fusaricidins, iturins with high sensitivity and accuracy in minimum time (Costa et al., 2022; Tsai et al., 2022).

Recently, there have been examples of the biological control of diseases caused by *N. dimidiatum*, mainly with biocontrol fungi and bacteria. For example, *Trichoderma harzianum* and *T. atroviride* reduced pathogen growth. Biocontrol bacteria such as *B. amyloliquefaciens*, *Penicillium rolfsii* and *B. subtilis* have also shown strong *in vitro* antifungal effects on *N. dimidiatum* (Ratanaprom et al., 2021; Wang et al., 2021). However, the inhibitory effect of biocontrol agents against *N. dimidiatum* has not been well elucidated to date, including the effects on the viability of conidia and ultrastructural modifications, or the ability to locate potential targets of conidia and mycelia. Therefore, in this study, we aimed to investigate the effectiveness of biocontrol microbes in suppressing pitaya canker. The objectives of this study were (1) to screen and identify effective biocontrol bacteria against pitaya canker; (2) to determine the biological control mechanisms of a biocontrol bacterium of *P. polymyxa*; (3) to clarify the potential capability of the bacterium for reduction of pitaya canker; and (4) to explore the molecular mechanism to identify the major target sites using RNA-seq. The results obtained in the present study provide promising information on bacterial biocontrol agents for controlling pitaya canker.

## 2. Materials and methods

### 2.1. Isolation of microorganisms

Soil samples (35) were collected from the 5–10 cm deep rhizospheres of seven pitaya orchards in Guangxi Province, China. To study the microorganisms present in these samples, 5 g of soil was added to autoclaved distilled water in a shaker for 30 min at 200 rpm at 28°C. Serial dilutions ranging from 10^−1^ to 10^−3^ were created from the supernatant, which was then spread over PDA plates (potato, 200 g; glucose, 20 g; agar, 20 g; ddH_2_O, 1,000 mL). After a 72 h incubation at 28°C, single morphological colonies were selected and purified on PDA plates by repeated streaking.

### 2.2. Screening of antagonistic bacteria against *Neoscytalidium dimidiatum*

To determine the potential of these isolates as antagonists, preliminary screening was conducted using the dual culture method (Ferreira et al., 1991). Phytopathogenic isolate BH5 was used in this test. BH5 was known to cause pitaya canker and was identified as *N. dimidiatum* by an internal transcribed spacer gene sequencing, with the NCBI GenBank database accession number OP393909.1. Analysis using BLAST (Basic Local Alignment Search Tool) revealed 99% sequence similarities of the strains with those of *N. dimidiatum* (Genbak accession number MZ047291.1 and OL455801.1). After demonstrating noticeable antagonistic activity against *N. dimidiatum*, isolates with inhibitory capacity were stored in 20% (v/v) glycerol at −80°C, and the strain with the strongest inhibition ability (labeled AF01) was selected for further studies.

### 2.3. Antifungal spectrum tests

A collection of pitaya fungal pathogens of *Fusarium equiseti* (C), *Botrytis cinerea* (D), *Bipolaris cactivora* (E) and *Gilbertella persicaria* (F) were tested (Lin et al., 2018). Inhibition of the pathogens was assessed as described: 1 μL of AF01 bacterial culture was inoculated 2.5 cm away from the edge of the plate twice, with three replicate plates. The antimicrobial activity was calculated by the percentage of mycelium growth inhibition compared to the control according to the formula: (R_1_ − *R*_2_)/R_1_ × 100, where R_1_ and R_2_ were the radii of the pathogen colony in the control and the antagonist colony, respectively.

### 2.4. Identification of strain AF01

Morphological characteristics of AF01 were observed on nutrient agar (NA). To determine the 16 s rDNA gene sequence of strain AF01, genomic DNA was extracted using the TIANamp Bacteria DNA Kit (TIANGEN, Beijing, China). The 16 s rDNA gene was amplified by PCR using the primers 27F:5′-AGAGTTTGATCCTGGCTCAG-3′; 1492R 5′-GGTTACCTTGTTACGACTT-3′. The 50 μL PCR mixture contained 25 μL 2 × Es Taq MasterMix (Dye), 2 μL 10 μM primers each, 2 μL genomic DNA, and 19 μL double distilled water. The PCR program was as follows: denaturation at 94°C for 2 min, 30 cycles at 94°C for 30 s, 55°C for 30 s, 72°C for 30 s, and final extension at 72°C for 2 min. The amplified PCR product was sequenced by Tsingke Biotechnology (Guangzhou, China), and the resulting sequences were subjected to BLASTN searches using the GenBank database. A phylogenetic tree was constructed using Phylosuit 1.2.2, based on the neighbor-joining method. The biochemical properties of strains AF01 was characterized using a GEN III MicroStation (BIOLOG, Hayward, CA, United States). A 150 μL bacterial suspension (10^8^ CFU/mL) was added to each well of a GEN III MicroPlate containing carbon source utilization assays and 23 chemical sensitivity assays. The MicroPlates were incubated for 14 h to form phenotypic fingerprint through the reduction of the tetrazolium redox dye caused by increased respiration. The phenotypic pattern in the GEN III MicroPlate was analyzed using read on the BIOLOG automated microbial analysis system software (GEN_ III_v2.8.0.15G).

### 2.5. Effects of cultural conditions on biomass and inhibitory activities of strain AF01 against *Neoscytalidium dimidiatum*

To test the ability to produce antimicrobial lipopeptides on different media, strain AF01 was grown in 100 mL PDB (the same components except agar), Landy (glucose, 20 g; glutamic, acid 5 g; yeast extract, 1 g; K_2_HPO_4_, 1 g; MgSO_4_, 0.5 g; KCl, 0.5 g; CuSO_4_, 1.6 mg; Fe_2_(SO4)_3_, 1.2 mg; MnSO_4_, 0.4 mg; ddH_2_O, 1,000 mL), NA (beef extract, 3 g; peptone, 5 g; glucose, 2.5 g; ddH_2_O, 1,000 mL), LB (tryptone, 10 g; yeast extract, 5 g; NaCl, 10 g; ddH2O, 1,000 mL), GSC (glucose, 20 g; starch, 20 g; (NH_4_)_2_SO_4_, 20 g; yeast extract, 10 g; K_2_HPO_4_, 2.6 g; FeSO_4_· 7H_2_O, 0.1 g; MgSO_4_· 7H_2_O, 0.5 g; NaCl, 0.25 g; CaCO_3_, 9 g; ddH_2_O, 1,000 mL), BPYDB (beef extract, 3 g; peptone 5 g; yeast extract, 1 g; glucose, 10 g; ddH_2_O 1,000 mL). After shaking at 28°C for 72 h, each culture was centrifuged at 10,000 rpm for 10 min, and supernatants were filtered through a 0.22 μm filter into Oxford cups. The Oxford cups were placed equidistantly in molten PDA medium (20 mL/dish, 9 cm flat dish). After solidification, the oxford cups were removed and the plates were evenly coated with 200 μL of *N. dimidiatum* spore suspensions (10^7^ CFU/mL). Then, each cell-free culture was added to the wells, and the diameter of the inhibition circle was measured by the crossover method after incubation for 72 h. Experiments in conical flasks cultivations were carried out in triplicate; mean value and standard deviation were calculated.

Strain AF01 was incubated in the optimal screening medium (1%, v:v) on a rotary shaker (180 rpm) at 28°C for different time courses ranged from 0 to 96 h. The OD_600_ values of the resultant cell suspensions were separately measured at each incubation time using the spectrophotometer. The inhibitory activities of the cell-free suspensions against *N. dimidiatum* at each incubation time were diluted into 1:10 with melted PDA. A *N. dimidiatum* plug (0.5 cm diameter) was placed at the center of a PDA plate and incubated at 28°C for 72 h and examined the inhibition rate, separately.

### 2.6. Effect of AF01 culture filtrate on hyphal growth of *Neoscytalidium dimidiatum*

*Paenibacillus polymyxa* AF01 was grown in a PDB liquid medium and incubated at 28°C, 180 rpm for 48 h, then the suspension was filtered through a 0.22 μm bacterial filter. Filtrate of AF01 was added to PDA at final concentrations of 2.5%, 5%, and 10%, and three plates were used for each treatment to examine the inhibition rate as 2.5. PDA plates without the AF01 culture filtrate were used as controls. A *N. dimidiatum* plug (0.5 cm diameter) was placed at the center of a PDA plate and incubated at 28°C for 72 h. The morphology of *N. dimidiatum* mycelia treated with AF01 culture filtrate was compared to that of mycelia untreated with AF01 under an optical microscope of OLYMPUS BX53 (OLYMPUS, Tokyo, Japan).

### 2.7. Effect of AF01 culture filtrate on spore germination of *Neoscytalidium dimidiatum*

Four days after inoculation, conidia were rinsed from *N. dimidiatum* dishes with 2.5%, 5%, and 10% AF01 culture filtrates to collect spores and adjusted to 10^7^ cells/mL by a hemocytometer. Control was washed with PDB only. Each treatment had three replicates. Conidia suspensions were incubated at 28°C, and spore germination was counted when the control germination rate was over 90%. Approximately a hundred *N. dimidiatum* conidia per replicate were examined microscopically for germination. To measure the bud lengths, line tool of cellSens (Ver.2.2) was used to draw a straight line from the germ neck to the germ tip. The measure of the length of this line was taken as the approximate length of the germ tube. The inhibitions of germination and germ tube elongation were then calculated using the formula: Inhibitions (%) of conidia germination = (average germinated conidia in control − average germinated conidia in treatment)/average germinated conidia in control × 100. Inhibitions (%) of germ tube elongation = (average germ tube length in control − average germ tube length in treatment)/average germ tube length in control × 100.

### 2.8. Assay of cell contents leakage

Conidia suspensions (~10^6^ cells/mL) of *N. dimidiatum* were grown in PDB media at 180 rpm for 3 days at 25°C. The mycelia and conidia were collected and washed three times with sterile distilled water, then resuspended in sterile distilled water containing 10% AF01 culture filtrate and shaken at 28°C for 0–6 h. The control was treated with 10% PDB. The supernatant was collected centrifugation at 6,000 rpm for 5 min. Then, the OD_260_ and OD_280_ values were detected to evaluate the leakage of nucleic acid and proteins from *N. dimidiatum* by NanoDrop2000 (Thermo, MA, United States). Electrolyte leakage was detected in a conductometer (LEICI, Shanghai, China).

### 2.9. MALDI-TOF MS analysis

The potential antifungal components in AF01 culture were detected and identified by MALDI-TOF MS as previously reported (Vater et al., 2015) with slightly modified. The culture of AF01 was centrifuged at 12,000 rpm, 10 min, and the supernatant was further filtered through a 0.22 μm bacterial filter to get the cell-free extraction of AF01, which was 10 times diluted with acetonitrile. Then 5 μL diluted cell-free extraction of AF01 was mixed with the same volume of matrix solution (a saturated solution of α-hydroxy-cinnamic acid in 50% aqueous acetonitrile containing 0.1% trifluoroacetic acid). The mixture (2.5 μL) was spotted on the target, air-dried, and measured. Mass spectra were obtained in positive ion and line modes with a QuanTOF MALDI-TOF mass spectrometer (IntelliBio, Shandong, China) equipped with a 337 nm nitrogen laser. For recording mass spectra in the range of 0.8 to 1.4 kDa, the focus mass was 1 kDa, the detector voltages were set at −0.44 kV, the laser pulse energy was set at 3.0 μJ, and each spectrum was recorded 800 times. The resulting mass/charge ratios (m/z) were used for component identification by comparing with the known fusaricidins.

### 2.10. Assembly screening for differentially expressed genes, gene ontology annotation, and Kyoto encyclopedia of genes and genomes enrichment

Strain *N. dimidiatum* BH5 was inoculated on a PDA plate with pre-placed cellophane and cultured for 36 h at 28°C. For the treatment, cell-free AF01 culture medium was diluted 1:10 with PDB, and 15 mL was added to plates co-cultured with *N. dimidiatum*. The control was treated with PDB alone. After 3 h, the AF01 culture filtrate was removed, and *N. dimidiatum* was washed thrice with sterile water. Treated and control *N. dimidiatum* mycelia were collected and subjected to intertranscriptomic analysis.

The TRIzol® Reagent (Plant RNA Purification Reagent) was used to extract total RNA from treated and control *N. dimidiatum* mycelia according to the manufacturer’s instructions (Invitrogen). DNase I (TaKaRa) was used to remove genomic DNA. The quality of RNA was verified by examining RNA degradation and contamination on 1% agarose gel. Then RNA quality was assessed using the 2,100 Bioanalyser (Agilent Technologies) and quantified using the NanoDrop2000. The sequencing library was only prepared from high-quality RNA samples with an OD_260_/ OD _280_ = 1.8–2.2, OD_260_/OD_230_ ≥ 2.0, RIN ≥ 8.0, 28S:18S ≥ 1.0, and > 1 μg. The Illumina HiSeq X Ten platform (Illumina, San Diego, CA, United States) was used to sequence the cDNA libraries prepared by Shanghai Majorbio Bio-Pharm Technology Co., Ltd., Shanghai, China. Alignment of reads was done to the reference genome of *N. dimidiatum* BH5 (unpublished). The raw reads were subjected to quality control (QC; Q30 > 99.9%). Raw data were filtered to obtain high-quality sequencing data (clean). Significantly differentially expressed genes (DEGs) were identified based on a threshold of |log2 (foldchange)| ≥ 1 and P-adjust ≤ 0.05 using DESeq2 software tools. To further understand the biological functions of the DEGs, functional enrichment analysis was performed using Gene ontology (GO) and the Kyoto Encyclopedia of Genes and Genomes (KEGG). The significance of the enriched GO terms and metabolic pathways were determined at adjusted *p* ≤ 0.05 compared to the whole-transcriptome background. GO functional enrichment and KEGG pathway enrichment analyses were performed using the software tools Goatools^1^ and KOBAS,^2^ respectively.

### 2.11. Pot experiment

Disease-susceptible pitaya (*Hylocereus polyrhizus*) “Jindu No. 1” was used in this experiment. The seedlings of pitaya were transplanted into pots, and after 3 weeks, the plants with 2–3 cladodes in a core, with similar growth status and no mechanical injury, were selected. Plants were disinfected with 75% ethyl alcohol for 1 min, followed by washing with sterile distilled water three times then air dried. Fermented bacterial suspension of AF01 (~5 × 10^7^ CFU/mL) was sprayed on the pitayas. Sterile water was used as the control. After 24 h, spore suspensions (~10^7^ cells/mL) of the pathogens were sprayed onto the cladodes. The experiment used a randomized block design with three replicates of two pots each; 73 twigs were tested. All plants were maintained in light growth chambers and then were incubated at 28°C and 80% humidity. Disease severity was rated after 14 and 21 days post infection (dpi) by using an index of 0–9 (0 = healthy; 1 = lesion area less than 5%; 3 = lesion area 6%–10%; 5 = lesion area 11%–25%; 7 = lesion area 26%–50%; and 9 = lesion area 51%–100%). The disease index (DI) was then calculated using the formula: Disease index = [Σ (rating × number of plants rated)/(total number of plants × highest rating)] × 100.

### 2.12. Field efficacy assessment of the AF01 against pitaya canker

To confirm the preventive effects of pitaya canker under field conditions, experiments were conducted in a field naturally infested with *N. dimidiatum* in September of 2021. The field tests were conducted at the Guangxi Academy of Agricultural Sciences experimental station in Nanning, China (108.063786°E, 23.252453°N, soil pH: 5.64, soil type: loam, organic matter: 29.2 g/kg) cultivated with “Jindu No. 1” pitaya plants. Pitaya cankers occur regularly every year at this station. The field trial was started on September 4 and finished on September 25 in 2021. At the start, a batch of very young fruit (approximately 1 week after flowering) appeared. Pitaya were grown in 2.5 × 0.5 m (row spacing × plant space) plots. The experimental design was a complete randomized block, and each treatment area was 15 m^2^, consisting of 30 plants per replicate with three replicates. During the trial, the climatic conditions recorded by an automatic weather station (Campbell, Logan, Utah, United States): rainfall 190.5 mm; rainy days: 8; temperature 23.13°C–38.54°C, daily average temperature: 25.30–31.23°C, and RH 40.16%–99.9%. Treatments of strain AF01 and 250 g/L pyraclostrobin EC (*BASF*) were set up in the trial. Strain AF01 was grown in PDB under agitation (180 rpm) at 28°C for 48 h, and liquid culture were diluted into 10 × and 20 × (1 × 10^8^ and 5 × 10^7^ CFU/mL, respectively) with H_2_O. Pyraclostrobin (166.7 mg kg^−1^, active ingredient) was separately sprayed on the plants to control pitaya canker. Clean water was sprayed on the control blocks. All treatments were carried out with battery-pressurized backpack sprayer with a hollow cone nozzle delivering at 0.2 KPa. Each plot was sprayed with 2.5 L of liquid per plot, equivalent to 1666.7 L per hectare. The application was performed three times at 7-day intervals. All pitaya fruits with no symptoms at the first treatment were signed in each repeating area to investigate disease incidence. The DI investigation was performed twice: 7 days after 2nd application time, when occurrence of pitaya canker was significantly at CK plots. And the final investigation was performed 7 days after the last application. Disease grading for pitaya canker was based on a pot experiment. The formation for calculating the disease index as 2.10 and control efficiency was as follows: Control efficiency = [(disease index of control area − disease index of treatment area)/disease index of control area] × 100.

### 2.13. Statistical analysis

Statistical analyses were performed in SPSS software version 26 (SPSS, Chicago, IL, United States), significant differences (*p* < 0.05) were analyzed by one-way ANOVA with Duncan’s multiple range test.

## 3. Results

### 3.1. Isolation of biocontrol bacteria

**Figure 1:**
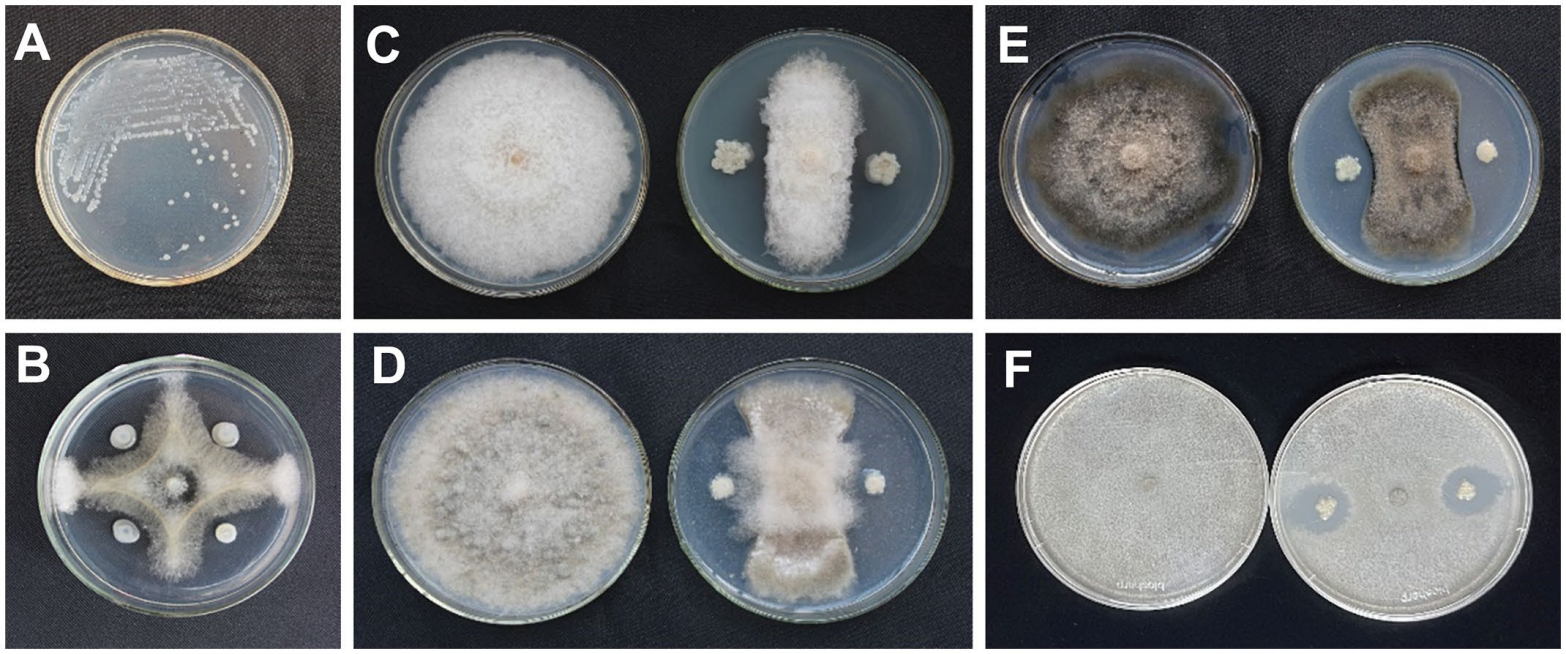
Colony morphology of *Paenibacillus polymyxa* AF01 on NA plate **(A)**. Inhibition effect of *P. polymyxa* AF01 on mycelial growth of *Neoscytalidium dimidiatum* **(B)**, *Fusarium equiseti* **(C)**, *Botrytis cinerea* **(D)**, *Bipolaris cactivora* **(E)**, and *Gilbertella persicaria* **(F)** based on a dual culture method.

A total of 113 bacterial isolates were collected from different sites, and five strains showed antagonistic activity against *N. dimidiatum in vitro*. The strain isolated from soil collected in Qinzhou, Guangxi Province, China, labeled AF01, showed the strongest antagonistic activity against *N. dimidiatum* with an inhibition rate of 76.80% (Figure 1B).

### 3.2. Inhibitory spectrum of strain AF01

Strain AF01 revealed strong inhibitory abilities against all the tested pathogens *F. equiseti*, *B. cinerea*, and *B. cactivora*. The highest percentage inhibition of radial growth (PIRG) value of 78.29% was observed for AF01 against *B. cinerea* (Figure 1D), and the lowest recorded, 24.80%, was observed against *G. persicaria* (Figure 1F). AF01 had a relatively weak inhibitory effect on *B. cactivora* (Figure 1E) and *equiseti* (Figure 1C), with 60.94% and 66.28% inhibition rates, respectively (Table 1).

**Table 1:** Assessment of AF01 antagonistic activity against four fungal pathogens *in vitro*.

Pathogens	Inhibition rate (%) ± SD
*Botrytis cinerea*	78.29 ± 0.67 a
*Fusarium equiseti*	66.28 ± 1.16 b
*Bipolaris cactivora*	60.94 ± 0.73 c
*Gilbertella persicaria*	24.80 ± 0.67 d

Values in the column indicate the mean ± standard deviation (SD) of the maximum/minimum radius of the pathogens of two repeated experiments. Values followed by different letters are significantly different according to Duncan’s multiple range test (*p* < 0.05) based on arcsine square-root transformed values of percentage.

### 3.3. Identification of bacterial isolates

**Figure 2:**
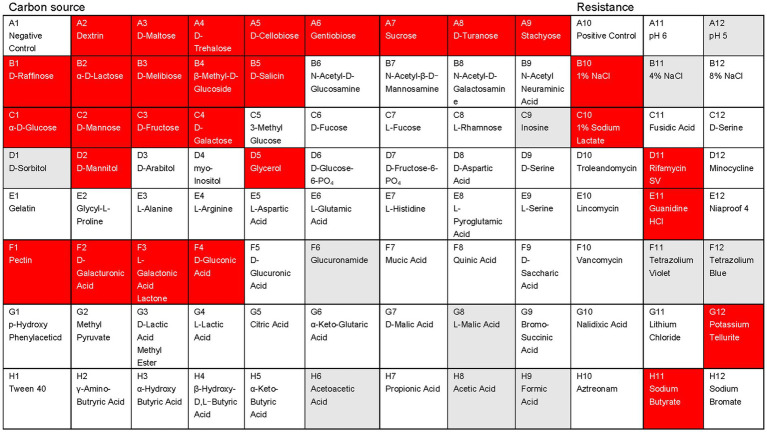
Physiological and biochemical characteristics analysis of the *Paenibacillus polymyxa* AF01using the Biolog GEN III MicroPlates system. Different patterns of 71 carbon sources and 23 susceptibility factors were observed in the studied microorganisms: red, positive; white, negative; gray, weak.

**Figure 3:**
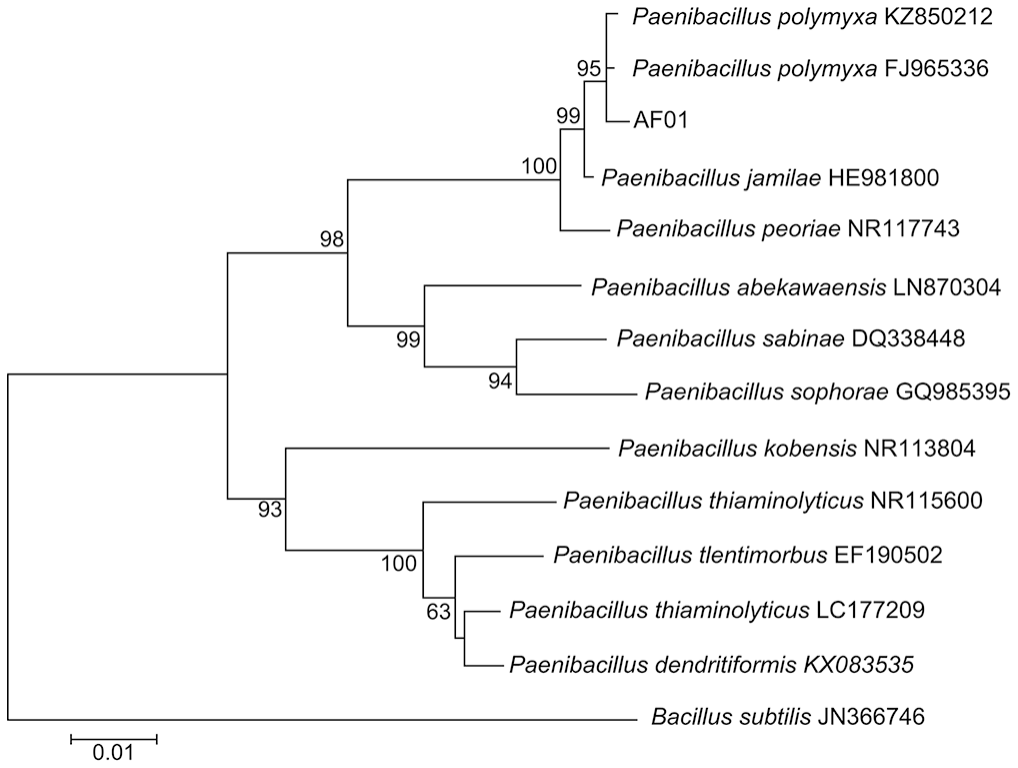
Neighbor joining phylogenetic tree showing the position of *Paenibacillus polymyxa* AF01 isolate with other species of *Paenibacillus* spp. and related taxa based on 16S rDNA gene sequences. Bootstrap values (expressed as percentages of 1,000 replications) are indicated at tree branch points.

AF01 is a rod-shaped Gram-negative bacterium that appears as a bulged surface of milky white, opaque colonies with irregular margins on an NA plate (Figure 1A). The beeswarm figure presented strains AF01 similar in the use of most carbon source substrates and assayed chemical compounds compared to the BIOLOG databases (Figure 2). Thereby, the strain AF01 was confirmed to be *Paenibacillus polymyxa* (SIM value:0.724, PROB value:0.910, and DIST value:2.878). In addition, BLAST analysis of the 16 s rDNA genes showed that the AF01 (GenBank database accession number: OQ601576) had an identity of 99% for *Paenibacillus polymyxa* (CP009909 and CP025957), suggesting that the strain was classified as *Paenibacillus* or *Paenibacillus polymyxa*. Phylogenetic analysis revealed the close clustering of strain AF01 with the sequences of *Paenibacillus* spp. Furthermore, AF01 and *P. polymyxa* were clustered together (Figure 3).

### 3.4. Effects of cultural conditions on biomass and inhibitory activities of strain AF01 against *Neoscytalidium dimidiatum*

**Figure 4:**
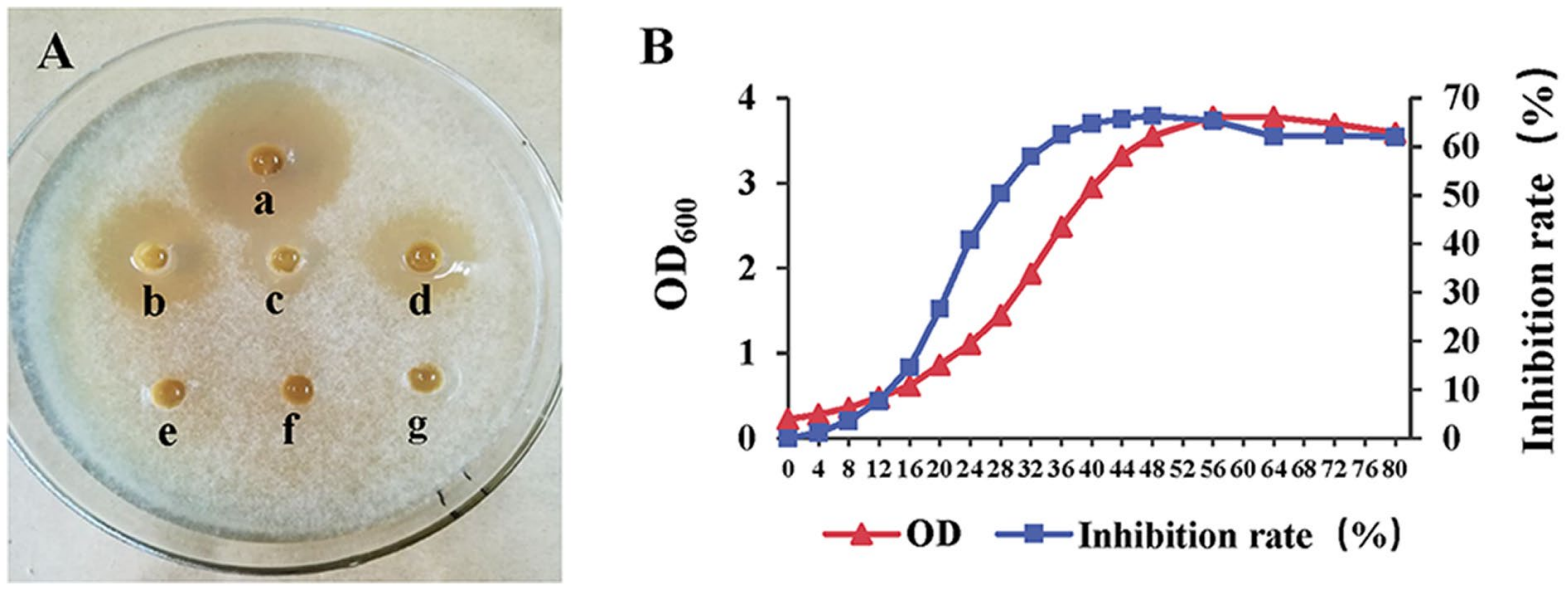
Agar well diffusion assay illustrating the growth inhibition of *Neoscytalidium dimidiatu*m by cell-free supernatants extracted from different media **(A)**. **(a)** PDB (inhibition zone 27.5 mm); **(b)** GSC (inhibition zone 2.24 mm); **(c)** Landy (inhibition zone 1.08 mm); **(d)** PBYDB (inhibition zone 1.86 mm); **(e)** NA (no inhibition zone); **(f)** LB (no inhibition zone); (g) ddH_2_O. Variation curves of cultural time on biomass (OD_600_) and inhibitory activity (inhibition rate) of strain AF01 against *N. dimidiatum* **(B)**. The OD_600_ values and inhibition rates were separately measured at each incubation time.

The inhibitory activity of strain AF01 against *N. dimidiatum* was detected in three of all media. The inhibition zone with the largest diameter (27.5 mm) were observed in PDB. The inhibit activities of strain AF01in GSC was higher than BPYDB and Landy. On the other hand, no inhibition zone was observed in LB and NA (Figure 4A). The bacterial biomass expressed by OD_600_ values and the inhibition rates of strain AF01 against *N. dimidiatum* obviously increased before 48 h of incubation and subsequently maintained at a stable level. The highest biomass (OD_600_ = 3.78) and peak inhibition rate (66.88%) occurred at the 56 and 48 h of incubation, respectively (Figure 4B).

### 3.5. Determination of antifungal activity of AF01

**Figure 5:**
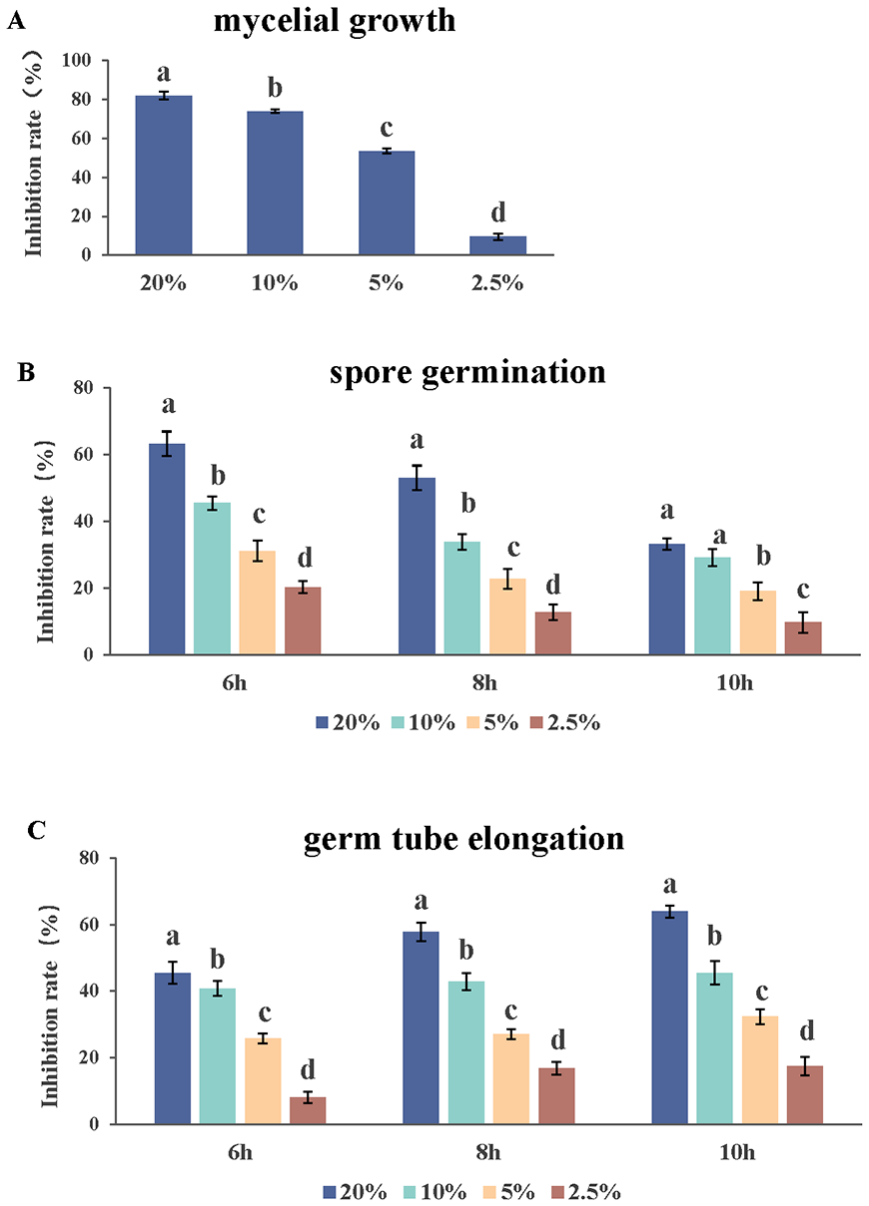
Effect of *Paenibacillus polymyxa* AF01 culture filtrate on three group of mycelial growth **(A)**, spore germination **(B)** and germ tube elongation **(C)** of *Neoscytalidium dimidiatum*. AF01 filtrate was applied at 20%, 10%, 5%, and 2.5%. Error bars indicate standard deviation. Different letters above the bars indicate significant difference within each group according to Duncan’s multiple range test (*p* = 0.05) based on arcsine square-root transformed values of percentage.

Under an optical microscope, two types of spores were observed in a ratio of about 1:1. There were significant differences in the antifungal abilities among the different application doses of the AF01 cultural filtrate in inhibiting *N. dimidiatum*. The AF01 cultural filtrate inhibited mycelial growth and spore germination by 9.47%–81.93% (Figure 5A) and 9.71%–62.22% (Figure 5B), respectively. Moreover, the inhibitions of germ tube elongation were 8.09%–63.97% (Figure 5C) at different treatments.

**Figure 6:**
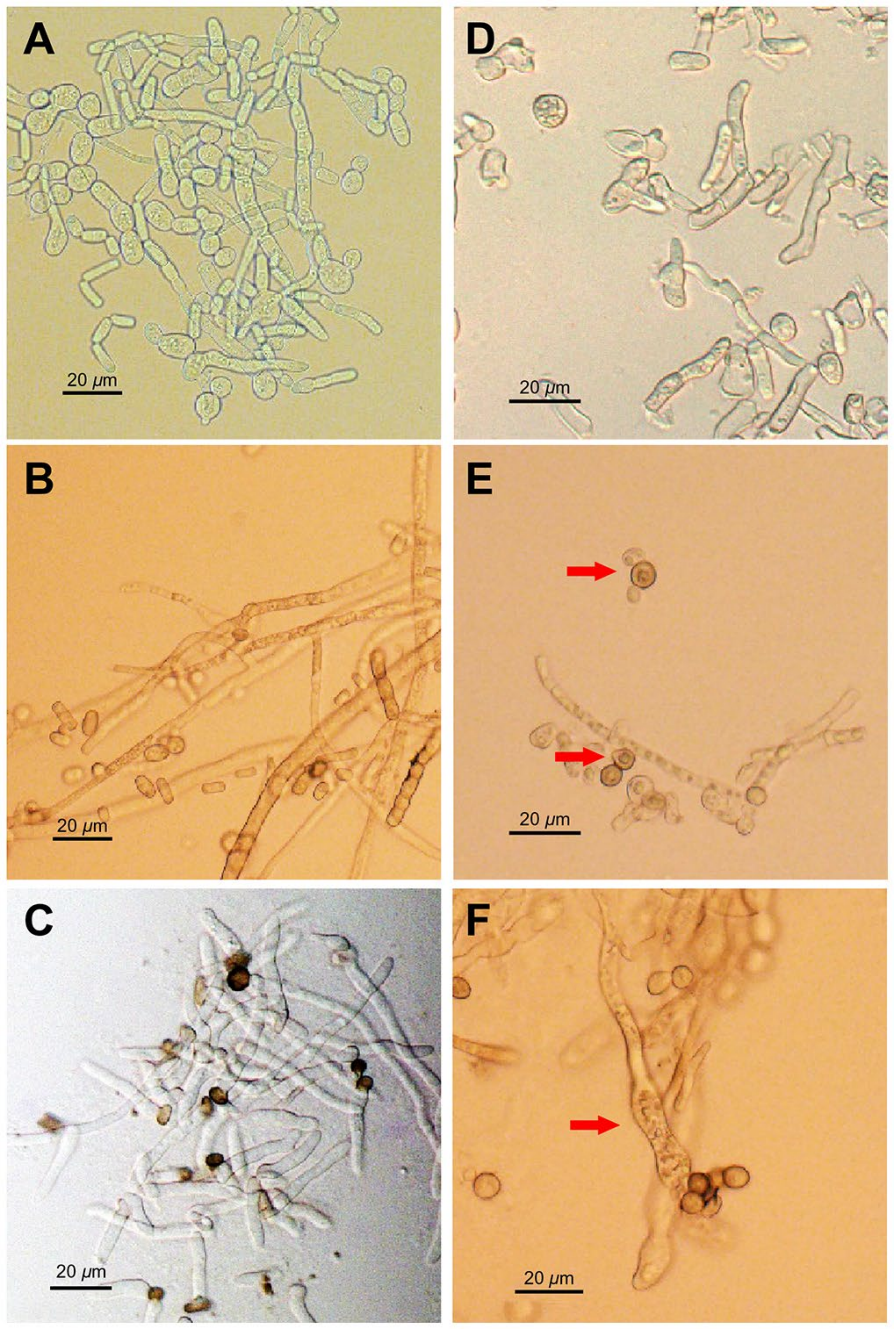
Inhibition effect of *Paenibacillus polymyxa* AF01 on phytopathogenic morphologies of *Neoscytalidium dimidiatum* observed via light microscopy. **(A–C)** The normal morphology in the control groups, and **(D–F)** the corresponding effect of *P. polymyxa*. Arrows indicate hyphal and conidia alterations with deformation **(D)**, vacuolar **(E)**, and swelling **(F)** of structures caused by *P. polymyxa* AF01.

Morphological changes in *N. dimidiatum* caused by AF01 culture filtrate were visualized using an OLYMPUS BX53 biological microscope at 600× magnification. The mycelia and germ tubes without AF01 cultural filtrate were normal and smooth but displayed marked structural changes when exposed to the AF01 cultural filtrate. Hyphae were severely deformed, with massive conglobation, uneven surfaces, expanded widths, whereas the germ tubes appeared shortened and swollen, and vacuolation also observed in conidia (Figure 6).

### 3.6. Assay of cell contents leakage

**Figure 7:**
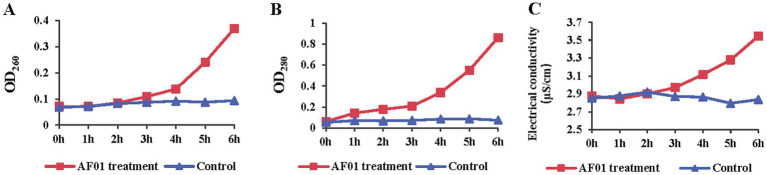
Membrane integrity change of *Neoscytalidium dimidiatum* in the presence of *Paenibacillus polymyxa* AF01 was evaluated by DNA leakage **(A)**, protein **(B)** and electrolytes **(C)**. *N. dimidiatum* was cultured with AF01 cultural filtrate. Supernatant were kept after centrifugation and quantified by absorption spectroscopy at 280 nm and 260 nm, respectively. Electrolyte leakage was detected with a conductometer.

Figure 7 shows the protein leakage assay used to determine cellular protein leakage. Once the bacterial cell membrane is irreversibly disrupted, cellular contents such as DNA, RNA, proteins and electrolyte leak out. For AF01-treated samples, the values of OD_260_ (Figure 7A), OD_280_ (Figure 7B) and electrical conductivity (Figure 7C) of the extracellular fluids increased rapidly, especially after 3 h, indicating cell membrane damage and the cell contents leakage.

### 3.7. Identification of antibiosis metabolites produced by strain AF01

**Figure 8:**
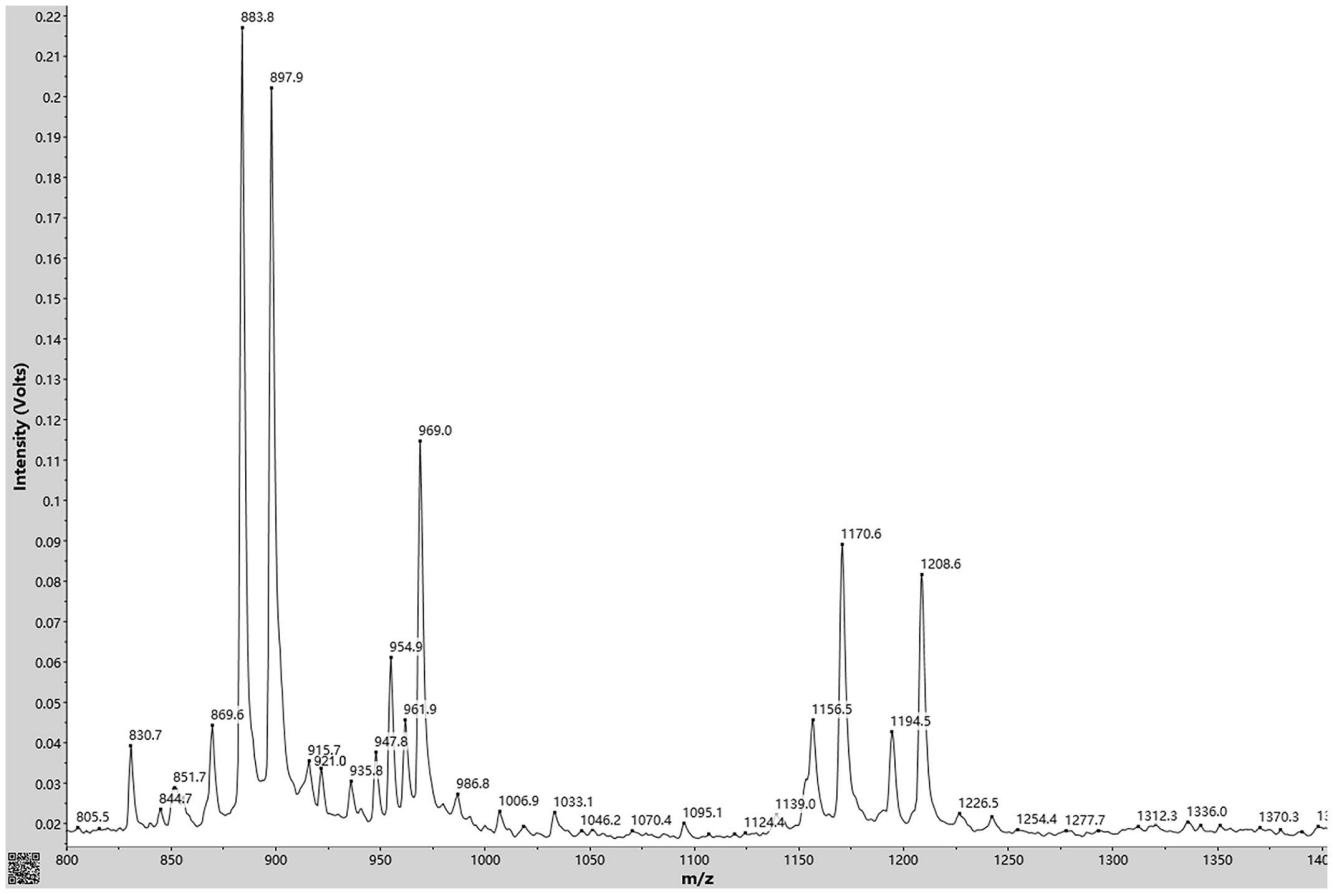
MALDI-TOF MS analysis of antimicrobial compounds produced by *Paenibacillus polymyxa* AF01 cultivated in PDB liquid medium. Mass peaks of fusaricidins were detected in the culture supernatant extracted with acetonitrile.

With simple sample preparation and high sensitivity, MALDI-TOF-MS has become a useful method for rapid *in situ* detection of complex families of natural compounds, such as fusaricidins and polymyxins. In present study, this technique was employed to identify the main ingredients of the AF0F culture. As shown in Figure 8, MALDI-TOF-MS spectrum of AF01 culture obviously exhibited the mass peaks of fusaricidins (m/z 860–1,000) and polymyxins (m/z 1,150–1,250). By comparing with the mass data of known fusaricidins the obtained mass peaks was identified as fusaricidins A-D (Table 2; Vater et al., 2015). According to available data, fusaricidins were found at m/z = 869.6 which could be derived from fusaricidin A by substitution of Thr in positions 4 with Ser. Two others yet unknown fusaricidins with molecular masses of m/z = 954.9 and 969.0 were related to fusaricidins A and B with a mass difference of 71 Da, indicating modification by attachment of an alanine residue (Mülner et al., 2021; Tsai et al., 2022). In addition to these identified mass peaks, there remain many peaks in the spectrum to be further identified, e.g., m/z 915.7, 921.0, 935.8, 986.8, 1006.9, 1033.1, and 1095.1.

**Table 2:** Fusaricidins detected in culture filtrates of *Paenibacillus polymyxa* AF01 by MALDI-TOF mass spectrometry.

[M + H, Na, K]^+^ m/z	Common name of ingredient	References
869.6		Vater et al. (2015), Mülner et al. (2021), and Tsai et al. (2022)
883.8	Fusaricidin A	Vater et al. (2015), Mülner et al. (2021), and Tsai et al. (2022)
897.9	Fusaricidin B; LI-F04b	Vater et al. (2015), Mülner et al. (2021), and Tsai et al. (2022)
915.7		Mülner et al. (2021)
921.0		Tsai et al. (2022)
935.8		Tsai et al. (2022)
947.8	Fusaricidin C; LI-F03a	Vater et al. (2015), Mülner et al. (2021), and Tsai et al. (2022)
954.9		Vater et al. (2015) and Mülner et al. (2021)
961.9	Fusaricidin D; LI-F03b	Vater et al. (2015), Mülner et al. (2021), and Tsai et al. (2022)
969.0		Vater et al. (2015), Mülner et al. (2021), and Tsai et al. (2022)
986.8		Tsai et al. (2022)
1006.9		Vater et al. (2015)
1033.1		Vater et al. (2015)

### 3.8. GO functions and KEGG pathway enrichment analysis of DEGs

**Figure 9:**
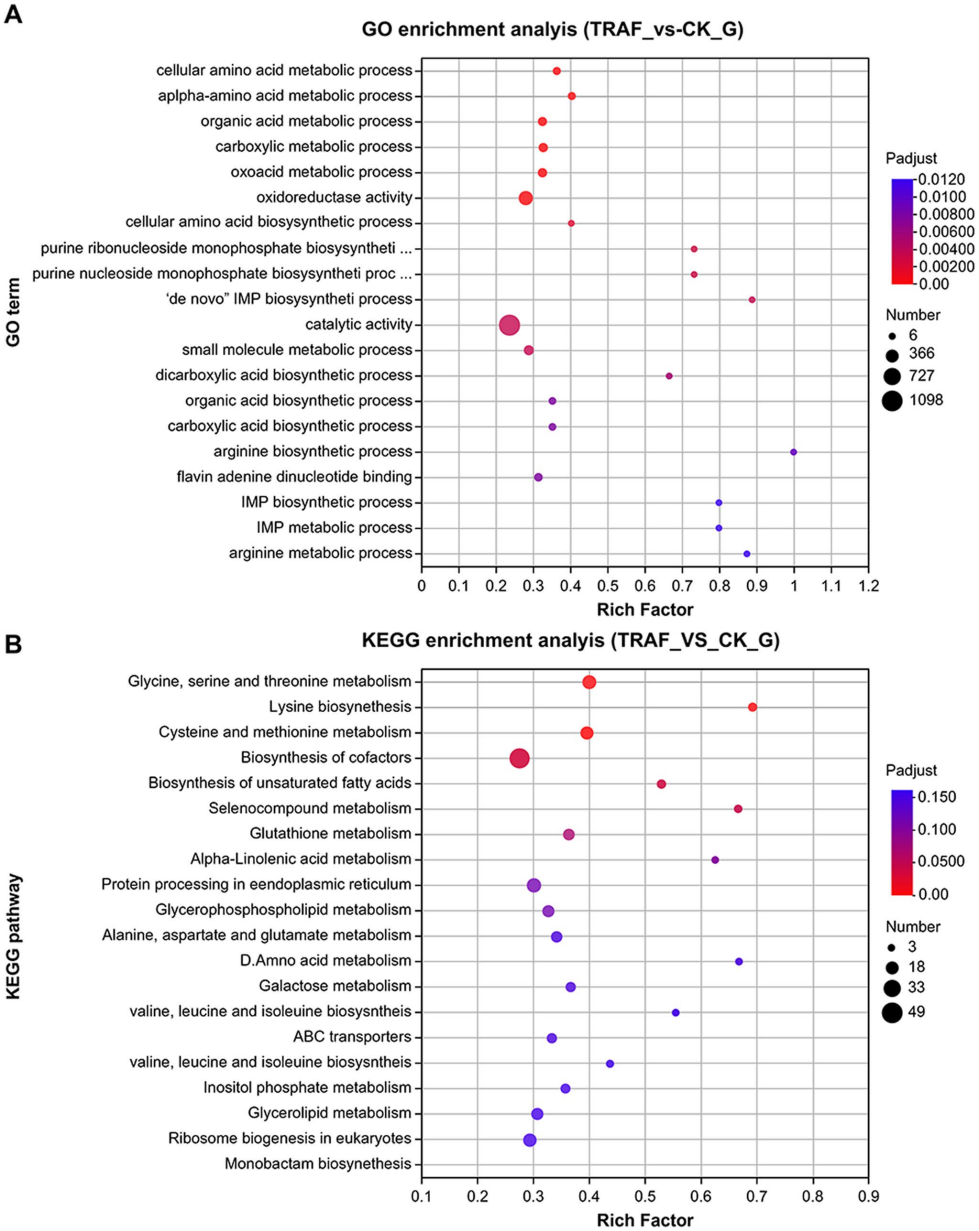
Top 20 terms of GO functional **(A)** and KEGG pathway **(B)** enriched with DEGs.

RNA-seq was used to analyze the transcriptomic response of *N. dimidiatum* BH5 after exposure to *P. polymyxa* AF01 for 3 h. Raw data were normalized and transformed into log2 values, and DESeq2 was used to analyze the differential gene expression of the samples. Subsequently, under co-cultivated conditions, strain BH5 exhibited significant differential gene expression with 2,248 genes, comprising 1,358 upregulated genes and 890 downregulated genes (|log2 FoldChange| ≥ 1). To understand the biological functions of the differentially expressed genes during co-culture, the annotated pathways of these genes were analyzed using the GO and KEGG databases. As a result, a total of 168 and 115 terms in the treatment group were enriched in the GO and KEGG, among which the top 20 terms of the GO and KEGG pathway are listed in Figures 9A,B (*p* < 0.05).

Compared to the control group, the genes involved in the biosynthesis of UDP-glucose, D-galacturonate, chitin, major structural components of fungal cell walls, and those related to pentose and glucuronate interconversions (ko00040, ko00520) were suppressed in response to *P. polymyxa* AF01 treatment for 3 h, particularly PLY (gene05693 and gene01489), which was downregulated by 3.92 and 3.56-fold, respectively (Table 3). Furthermore, the expression of genes associated with lipid metabolism (ko01040, ko00564, and ko00590) was prominently reduced following exposure to AF01, and some genes showed lower expression levels, such as GDE1 (gene08147), EPHX2 (gene03244), glpQ and ugpQ (gene09638), by more than 6-fold. These genes have been described as encoding hypothetical proteins, glycerophosphoryl diester phosphodiesterases, and alpha/beta hydrolase folds (Table 3). During protein processing in the endoplasmic reticulum, 25 genes exhibited differential expression. Of these, 23 were downregulated and two were upregulated. The downregulated factors play important roles in protein recognition, glucosylation, protein transport, glucosyl transfer, and receptor binding, as shown in Table 3.

**Table 3:** The expression of genes in cell wall metabolism, membrane metabolism, and protein processing in the endoplasmic reticulum in *Neoscytalidium dimidiatum* BH5.

Gene id	Gene description	Gene name	Log_2_FC(AF01/CK)
Cell wall metabolism			
gene02271	UTP-glucose-1-phosphate uridylyltransferase	UAP1	−1.4018
gene04405	Fungal chitin synthase	CHS1	−1.1220
gene09272	Nucleotidyl transferase	GMPP	−1.4269
gene09178	UTP-glucose-1-phosphate uridylyltransferase	UGP2, galU, galF	−1.1441
gene04109	putative endochitinase 1 precursor protein	E3.2.1.14	−1.0810
gene05693	Pectate lyase catalytic	UAP1	−1.8772
gene07098	Endopolygalacturonase	PLY	−1.4216
gene10699	putative dimeric dihydrodiol protein	E3.2.1.15	−1.0055
gene09178	UTP-glucose-1-phosphate uridylyltransferase	DHDH	−1.1441
gene07596	Transcription factor	UGP2, galU, galF	−1.1785
gene01489	Pectate lyase catalytic	LARA	−1.7511
gene02261	Fructose and mannose metabolism	PLY	−1.5771
Membrane metabolism			
gene05693	Pectate lyase catalytic	PLY	−1.8772
gene07098	Endopolygalacturonase	E3.2.1.15	−1.4216
gene10699	putative dimeric dihydrodiol protein	DHDH	−1.0055
gene09178	UTP-glucose-1-phosphate uridylyltransferase	UGP2, galU, galF	−1.1441
gene07596	Transcription factor	LARA	−1.1785
gene01489	Pectate lyase catalytic	PLY	−1.7511
gene00644	hypothetical protein	LOA1	−1.0552
gene00041	FAD-dependent glycerol-3-phosphate dehydrogenase	glpA, glpD	−1.6343
gene06725	nte family protein	TGL4	−1.6944
gene09638	Glycerophosphoryl diester phosphodiesterase	E3.1.4.46, glpQ, ugpQ	−2.7393
gene03155	putative phospholipase d active site motif protein	PLD1_2	−2.0977
gene10670	Phosphatidic acid phosphatase type 2/haloperoxidase	DPP1, DPPL, PLPP4_5	−1.4374
gene08147	hypothetical protein	GDE1	−2.7918
gene03244	Alpha/beta hydrolase fold-1	EPHX2	−2.5962
gene06725	nte family protein	TGL4	−1.6944
gene09236	putative 15-hydroxyprostaglandin dehydrogenase [NAD(+)] protein	HPGD	−1.3362
Protein processing in the endoplasmic reticulum			
gene07142	Heat shock protein Hsp20	HSP20	−1.4227
gene10617	Ribophorin I	OST1, RPN1	−1.1253
gene08060	putative ubiquitin-protein ligase sel1 protein	SEL1, SEL1L	−1.1990
gene07540	putative er-associated proteolytic system protein	DERL2_3	−1.2089
gene08246	Heat shock protein DnaJ	DNAJC3	−1.0853
gene04507	Glycoside hydrolase family 47	MAN1A_C, MNS1_2	−2.3405
gene02455	Protein transport protein sec61 alpha protein	SEC61A	−1.4032
gene04345	Translocation protein Sec62	SEC62	−1.1972
gene04131	Zinc finger RING-type protein	SYVN1, HRD1	−1.1323
gene10426	UDP-glucose:Glycoprotein Glucosyltransferase	HUGT	−1.6715
gene10415	hypothetical protein	SCJ1	
gene06723	Ribophorin II	SWP1, RPN2	−1.2449
gene07034	putative dolichyl-di-phosphooligosaccharide-protein glycotransferase protein	WBP1	−1.2131
gene08171	Oligosaccaryltransferase	OST4	−1.4953
gene03198	putative ubiquitin-conjugating enzyme e2 j1 protein	DERL2_3	−1.3057
gene03253	Glycoside hydrolase family 31	UBE2J1, NCUBE1, UBC6	−1.6264
gene01339	Glycoside hydrolase family 63	GANAB	−1.1708
gene00684	Legume-like lectin	MOGS	−1.2489
gene09548	Dad family protein	LMAN2, VIP36	−1.2223
gene07616	Heat shock protein Hsp70	OST2, DAD1	−1.6040
gene05581	Mannose-6-phosphate receptor binding protein	GANAB	−1.7727
gene09248	Heat shock protein Hsp70	PRKCSH	−1.2888

In addition, other genes downregulated in the treatment group were enriched in KEGG terms, including biosynthesis of cofactors, meiosis, chromatin structure and dynamics, peroxisomes, and the MAPK signaling pathway.

### 3.9. Efficacy of AF01 in controlling pitaya canker in pots

**Figure 10:**
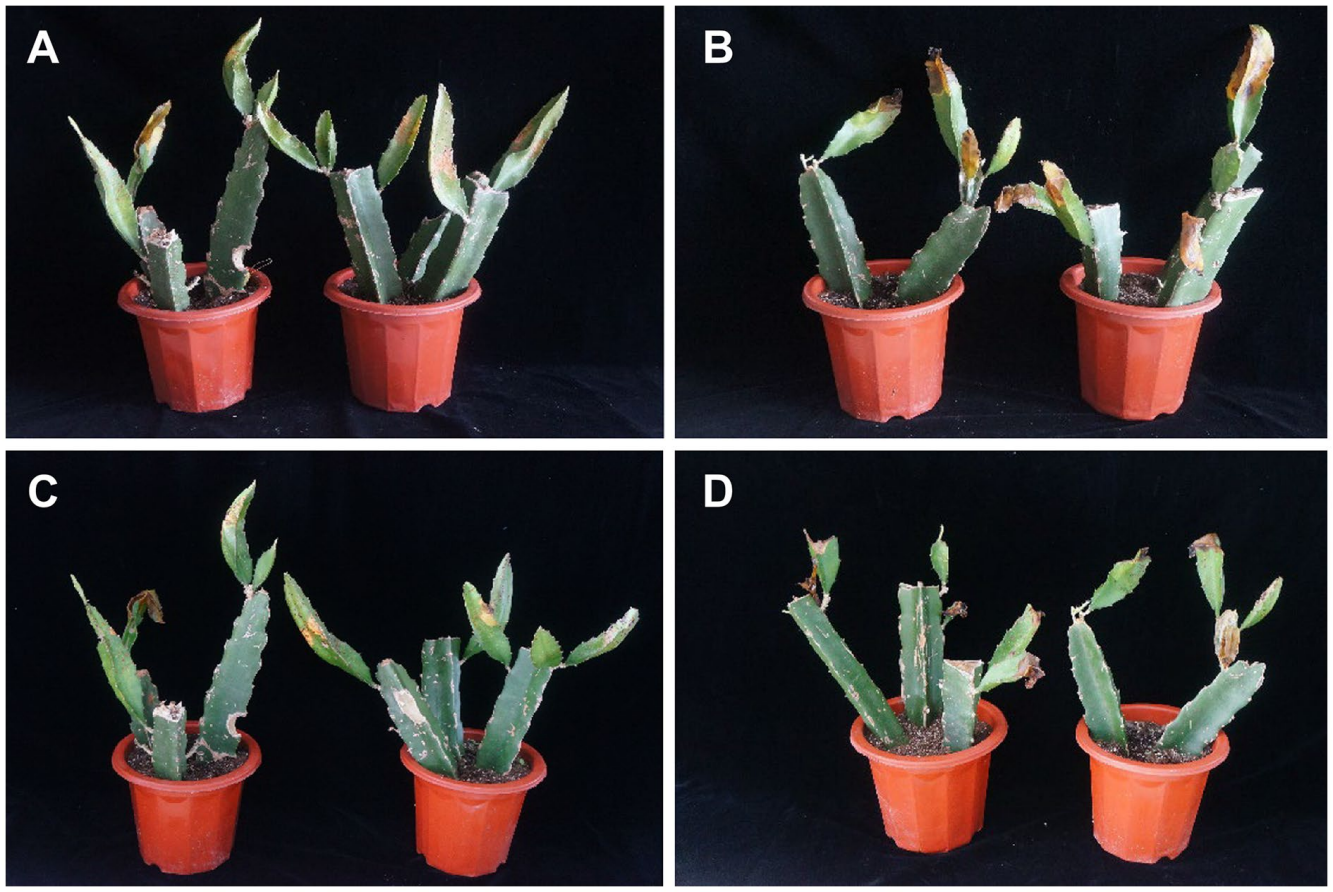
Symptom development in different groups of *Neoscytalidium dimidiatum* inoculation. Treatment: plants challenged with *Paenibacillus polymyxa* AF01 for 24 h and then with *N. dimidiatum*; control: plants challenged with *N. dimidiatum.* The pictures are representative of two independent experiments: treatment on the 14th day after *N. dimidiatum* inoculation **(A)**; treatment on the 21th day after *N. dimidiatum* inoculation **(C)**; control on the 14th and 21th day after *N. dimidiatum* inoculation **(B,D)**.

Disease suppression in pitaya was investigated to test the capacity of AF01 as a biocontrol agent. Typical symptoms of chlorotic spots appeared 10 days after inoculation with *N. dimidiatum*. The DI at 14 dpi reached 71.37 with control. By comparison, the *P. polymyxa* AF01 and pathogen treatment was 37.11, significantly lower than the foliage with only *N. dimidiatum* (Figures 10A,B). With age, severe canker symptoms, including necrosis, soft rot, and defoliation gradually appeared in the plants. At 21 dpi of inoculation, the DI of plants inoculated with *N. dimidiatum* was 81.97; however, in plants inoculated with only *P. polymyxa* AF01 and *N. dimidiatum,* was 50.43 (Figures 10C,D), suggesting a positive effect in controlling pitaya canker in this experiment (Table 4).

**Table 4:** Assessment of AF01 antagonistic activity against four fungal pathogens *in vitro*.

Treatment	Disease index (%) ± SD	
	14dpi	21dpi
AF01+ *Neoscytalidium dimidiatum*	37.11 ± 2.13 a	50.43 ± 6.16 a
*N. dimidiatum*	71.37 ± 3.50 b	81.97 ± 4.28 b

Values in the column indicate the mean ± standard deviation (SD) of AF 01 liquid culture for reduction of pitaya canker 14 and 21 days after inoculation with *N. dimidiatum*. Values followed by different letters are significantly different according to Duncan’s multiple range test (*p* < 0.05) based on arcsine square-root transformed values of percentage.

### 3.10. Field efficacy assessment of the AF01 against pitaya canker

*Paenibacillus polymyxa* AF01 fermentation broth reduced pitaya canker caused by *N. dimidiatum* in field experiments. The fungicide treatment had the greatest effect of 74.82%. The efficacies of different concentrations (5 × 10^7^ CFU/mL and 1 × 10^8^ CFU/mL) of AF01 suspension were evaluated and shown to be 50.68 and 65.28%, respectively. The control efficacy increased as the application frequency increased; after three treatments, 10^7^ CFU/mL, 10^8^ CFU/mL AF01, and pyraclostrobine suppressed pitaya canker with efficacies of 57.50%, 68.66%, and 79.70%, respectively (Table 5). The results showed that AF01 fermentation broth provided preservative effects by reducing chlorotic spots, suberization, and pitaya decay.

**Table 5:** Control effect of AF01 against pitaya canker in the field.

Treatment	7 days after the 2nd treatment		7 days after the 3rd treatment	
	Disease index	Control effect (%) ± SD	Disease index	Control effect (%) ± SD
10^7^ CFU/mL AF01	7.26	50.68 ± 4.67 b	9.31	57.50 ± 4.89 c
10^8^ CFU/mL AF01	5.11	65.28 ± 4.58 a	6.84	68.66 ± 4.51 b
250 g/L Pyraclostrobin EC	3.63	74.82 ± 4.40 a	4.36	79.70 ± 3.83 a
CK	14.62	-	21.66	-

Values in the column indicate the mean ± standard deviation (SD) of *Paenibacillus polymyxa* AF 01 liquid culture for control effect of pitaya canker after 2 or 3 treatments. Values followed by different letters are significantly different according to Duncan’s multiple range test (*p* < 0.05) based on arcsine square-root transformed values of percentage.

## 4. Discussion

Pitaya is highly susceptible to microbial diseases, particularly those caused by *N. dimidiatum* (Pan et al., 2021). Therefore, developing an effective prevention and control strategy to control this disease during production is crucial and necessary. As humans become increasingly concerned about food safety and environmental pollution, biocontrol has become increasingly important in sustainable agriculture and industrial biotechnology over time (Gao et al., 2019; Xu et al., 2020). Paenibacillaceae are found in the rhizosphere of different crops and found in marine sediments, forest plants, and insect larvae. In the context of the current information, *Paenibacillus* spp. can benefit agriculture through antimicrobial activity, nitrogen fixation, and phosphate solubilization and strongly affected plant-parasitic nematodes, oomycetes, pathogenic bacteria, and fungal phytopathogens, including *Fusarium oxysporum*, *B. cinerea,* and *Verticillium dahlia* (Grady et al., 2016). Multiple anti-fungal activity mechanisms are influenced by antibiosis, competition for nutrients and biological niches, heavy parasitism, and the induction of plant resistance (Zhai et al., 2021). In our study, among the 113 bacterial strains isolated from rhizosphere samples, strain AF01 from Qinzhou, Guangxi Province, China, showed the strongest antagonistic activity against *N. dimidiatum*, *F. equiseti*, *B. cinerea*, *B. cactivora,* and *G. persicaria*. Combined with the 16 s rRNA result, AF01 has a 99% identity with that of *P. polymyxa* (CP009909 and CP025957). According to current studies, *Paenibacillus* is one of the eight genera in the family Paenibacillaceae and currently comprises ~200 species (Grady et al., 2016). Comparative 16S rRNA sequence analysis could not demonstrate that the genus *Paenibacillus* consisted of *P. polymyxa* and close relatives. The identification of microbial taxonomy requires a combination of physiological, biochemical and molecular techniques (von der Weid et al., 2000). In this study, the strain AF01 was identified as *P. polymyxa* based on the results obtained from GEN III MicroPlate tests, where most biochemical characteristics of strain AF01 were consistent with those of *P. polymyxa*. Based on biochemical characters and the phylogenetic tree, strain AF01 was classified as *P. polymyxa.*

*In vitro* effects of *P. polymyxa* AF01 on the mycelial growth, spore germination and germ tube elongation provided the first evidence related to their potential toxicity and efficacy against the pathogenic *N. dimidiatum*. Compared with spore germination and germ tube elongation, hyphae growth phase was more sensitive to the effect of AF01 culture filtrate, while germ tube growth was less affected, which is in agreement with other findings on the antifungal activity of *P. polymyxa* and other *Paenibacillus* spp. against fungal species such as *Cylindrocarpon destructant*, *Rhizoctonia solani*, *Fusarium* sp., *Sclerotinia sclerotiorum*, *Pythium ultimum* (Bae et al., 2004), *Colletotrichum gloeosporioides* (Ran et al., 2021), *Pseudoperonospora cubensis*, *B. cinerea*, *Phytophthora capsica* (Kim et al., 2009) and *Magnaporthe oryzae* (Ali et al., 2021). Microscopic observations revealed that *P. polymyxa* AF01 hampered the fungus spore causing mycelial tip enlargement, protoplast cleavage and abnormal morphological alternations of the spores. A marked enhancement of fungal cell permeability was reflected by the abnormal and massive spillage of nucleic acid, proteins and electrolyte from macromolecular cytoplasmic components owing to cell damage by *P. polymyxa* AF01. Dispersal of conidia are the origin of many secondary infections on pitaya. The antifungal effects of AF01 on fungal spore germination may play a role in disease control, as the pathogen life cycle may be disrupted.

Fusaricidins produced by *P. polymyxa* strains play an important strategy for controlling plant pathogenic. These compounds comprised a guanidinylated β-hydroxy fatty acid and a cyclic hexapeptide. *Paenibacillus* stains produced various types of fusaricidins (Gil et al., 2021), where enormous diversity exists even within those found in the same bacterial. MALDI-TOF MS is a practical technique for detecting secondary metabolites without additional purification, allowing the identification of active compounds in crude extracts via mass spectrometry. More than 20 variants of these compounds have been detected and characterized in detail in *P. polymyxa*-M1, including fusaricidins A–D, LI-F03, LI-F04, LI-F05, LI-F06, LI-F07, and LI-F08 and other novel fusaricidin derivatives (Vater et al., 2015). Using a similar method, we demonstrated that AF01 produced 13 forms of fusaricidins, showing a much higher complexity than expected. Among which, fusaricidin A and fusaricidin D were the main fusaricidin found. Inhibitions of mycelial growth, spore germination, germ tube elongation caused by AF01 culture filtrate were observed in the study. Furthermore, this inhibiting action maybe due to fusaricidins, which cause cytoplasm leakage such as intracellular nucleic acid, proteins and electrolyte. The finding was consistent with reports that the main mode of fusaricidin against bacteria is due to the disruption of membrane integrity.

In this study, *N. dimidiatum* BH5 mycelia and spores were damaged and had a rough, swollen, and large area of cavitation that even appeared in the center of the cytoplasm after treatment with *P. polymyxa* AF01, which was probably due to damage to cell function. Based on previous studies that used ultrastructural observations and proteomic analyses, the synthesis of secondary metabolites involves multiple pathways as well as the inhibition of lipid, carbohydrate, and amino acid metabolism, thiamine biosynthesis, energy metabolism, and reduced reactive oxygen species (ROS) scavenging capacity (Han et al., 2017; Mokhtar et al., 2020). Our results suggest that the efficacy of *P. polymyxa* against *N. dimidiatum* BH5 is mediated by its ability to selectively block critical cellular processes including the transcription and translation mechanisms as well as RNA and DNA structural dynamics, energy production and conversion, and signal transduction pathways. Additionally, it appears to affect cell walls, membrane permeability, and protein biosynthesis.

Fungal cells possess dynamic cell walls that protect them against environmental stress and changes in osmotic pressure, making them critical to the species biology and ecology (Gow et al., 2017). Cell membranes serve a similar function, controlling the movement of substances in and out of the cell, making them functionally important in biological systems (Kraft, 2013). However, changes in these structures can cause cell wall and plasma membrane dysfunction, resulting in the leakage of protoplasm from conidia and mycelia and the formation of vacuoles in spores and mycelia. Therefore, cell walls and plasma membranes are major targets of antifungal drugs and volatile compounds because of their crucial roles in fungal cell survival (Bakkali et al., 2008). Similar to the effect of iturin A on *Phytophthora infestans* and fusaricidin on *Bacillus subtilis* and *Fusarium moniliforme* (Han et al., 2017), RNA-seq results indicated that the expression levels of CHS1, galU, galF, PLY, glpA, glpD, glpQ, ugpQ, GDE1, MVK genes were significantly altered by *P. polymyxa* AF01 treatment. This might adversely affect the biosynthesis of Glu, Rha, Gal, glycerone-P, sterol precursor, 1,2-diacyl-glycerol-3p, arachidonic acid significantly and thus suppressed the growth of the *N. dimidiatum*.

The endoplasmic reticulum (ER) is an organelle responsible for metabolism, signal transduction, and the growth and development of organisms. *P. polymyxa* AF01 treatment inhibited the expression of MAN1A-C, MNS1-2, HUGT, GANAB, and PRKCSH, resulting in significant disruptions in the accurate folding, post-translational modification, and final assembly of the cellular proteome of *N. dimidiatum* BH5. It may also reduce the secretion of sufficient quantities of cell-wall-degrading enzymes, proteases, pectinases, and other enzymes required to penetrate plant surfaces and cause necrosis (Joubert et al., 2011).

ROS are highly reactive, can damage cellular components such as proteins, lipids, and nucleic acids, and harm various biomolecules within biological systems (Scott and Eaton, 2008; Yang et al., 2022). Accordingly, peroxiredoxin and catalase T are significantly induced to detoxify ROS, maintain the redox balance, and resist oxidative damage to the cells (Hossain et al., 2015). This study examined the effects of *P. polymyxa* AF01 on *N. dimidiatum* BH5 cells. Treatment with *P. polymyxa* AF01 to pitaya resulted in the upregulation of ACOX1, ACOX3, DAO, and AAO, whereas MPV17 was downregulated, suggesting that the treated cells accumulated ROS. These alterations result in changes in the permeability of the fungal membrane, impaired membrane transport and respiration, and the disintegration of the fungal cell wall.

Results of the pot experiment strongly supported that *P. polymyxa* AF01 significantly reduces the severity of pitaya canker. However, the actual agricultural field is far more complex than the pot experiment under laboratory conditions. Further field studies were carried out to verify the control effect as the disease control efficacy of strain AF01 reached 68.66%. Despite being less potent than chemically synthesized chemical fungicides, *P. polymyxa* AF01 has the benefit of being more environmentally friendly and conforming to the idea of sustainable development. To improve its biological control effect, optimal application rate, timing, and frequency of AF01 are also important issues that must be considered in field production.

Although *P. polymyxa* can improve plant defense abilities to reduce disease occurrence; our previous study showed that irrigating with AF01 did not significantly reduce pitaya canker. it is not yet clear whether AF01 can regulate defensive response to *N. dimidiatum* infection. Therefore, studies in this field should be conducted in further work.

## 5. Conclusion

Pitaya canker is a fungal disease threatening the pitaya fruit industry. *P. polymyxa* AF01, a bacterium exhibiting potent antifungal activity against the pathogen *N. dimidiatum*, was identified using a combinate analyses. The main antifungal products of *P. polymyxa* AF01 are fusaricidins, which possess a broad antibacterial spectrum. Based on our existing data and previous studies, a hypothesis was proposed regarding the mode of fusaricidin produced by *P. polymyxa* in suppressing pitaya canker: it was postulated that fusaricidins disturbed cell wall biosynthesis, changes in membrane permeability direct inhibitory effects on spore germination and hyphal growth of *N. dimidiatum*. Considering its high potential as a biocontrol agent, additional studies are warranted to explore the development and practical applications of *P. polymyxa* AF01.

## Data availability statement

The datasets presented in this study can be found in online repositories. The names of the repository/repositories and accession number(s) can be found at: https://www.ncbi.nlm.nih.gov/, PRJNA943517.

## Author contributions

SL: conducting the experiments and writing the manuscript. XC: participating in experiments and revisions. LX: supervision. YZ: data and bioinformatics analyses. FZ: data curation. YL, LR, and XQ: participating in the design and experiments. JW: funding acquisition and writing-reviewed and edited. All authors contributed to the article and approved the submitted version.

## Funding

This work was supported by Guangxi Natural Science Foundation Youth Fund (grant number: 2017GXNSFBA19823).

## Conflict of interest

The authors declare that the research was conducted in the absence of any commercial or financial relationships that could be construed as a potential conflict of interest.

## Publisher’s note

All claims expressed in this article are solely those of the authors and do not necessarily represent those of their affiliated organizations, or those of the publisher, the editors and the reviewers. Any product that may be evaluated in this article, or claim that may be made by its manufacturer, is not guaranteed or endorsed by the publisher.

## Footnotes

^1^ https://github.com/tanghaibao/Goatools

^2^ http://kobas.cbi.pku.edu.cn/home.do
